# Trends in the Second Law of Thermodynamics

**DOI:** 10.3390/e25091321

**Published:** 2023-09-10

**Authors:** Roberto Zivieri

**Affiliations:** Istituto Nazionale di Alta Matematica (INdAM), Piazzale Aldo Moro 5, 00185 Rome, Italy; roberto.zivieri@unife.it

The Second Law of Thermodynamics represents a milestone in the history of not only physics but also chemistry, engineering, and, more generally, life and natural sciences. It has been known for over 150 years and is usually thought of as one of the most authoritative laws. It can be regarded as the completion of the First Law of Thermodynamics, which establishes the conservation of energy in thermodynamic systems as the counterpart to the conservation of mechanical energy in mechanical systems. It is a fundamental law of the universe and its universality can be proven by demonstrating its equivalence across all types of thermodynamic systems. To strengthen its generality and impact, it is sometimes elevated to the level of a principle and named the Second Principle of Thermodynamics [1,2,3], providing it with a philosophical nature. However, unlike other principles in physics that are viewed as unprovable postulates, it is empirical and has been rigorously proved. This law is generally applied to closed thermodynamic systems, physical systems in which the exchange of heat is allowed but the exchange of matter with the surrounding is not. It can be applied also to isolated thermodynamic systems where no exchange of heat or matter is allowed. As occurs for the First Law of Thermodynamics, it can be regarded as a general consequence of Newton’s laws of motion [4].

There are different formulations of the Second Law of Thermodynamics. The oldest one dates back to the 19th century and can be regarded as its classical formulation. It was enunciated in different forms. The first one is based on the Kelvin–Planck and Clausius statements [5,6,7,8,9,10,11], dealing with the exchange of heat and work in heat engines. Specifically, the Kelvin–Planck statement [5,6] reads “*It is impossible to construct an engine which, operating in a cycle, will produce no effect other than the extraction of heat from a reservoir and the performance of an equivalent amount of work*”. In other words, it is not possible for a heat engine absorbing heat from an external reservoir at a higher temperature *T*_H_ to convert all the heat into work without rejecting part of the absorbed heat to an external reservoir at a lower temperature *T*_L_ different from zero. Note that this restriction between a form of energy represented by heat *Q* and work *W* is not envisaged by the First Law of Thermodynamics. Indeed, according to the First Law of Thermodynamics, the equality *Q* = *W* realizes during isothermal transformations at a fixed temperature *T*, establishing an identity between energy and work, as occurs in mechanical systems. This means that the First Law of Thermodynamics, applied to closed thermodynamic systems and regarded as a law of conservation of energy in thermodynamic systems, which may gain or lose internal energy depending on the balance between heat and work, is less restrictive than the Second Law of Thermodynamics. Indeed, this latter law imposes a constraint on energy conversion, the exchange of heat, and its transformation into work. Moreover, the Second Law of Thermodynamics cannot be directly deduced from the First Law of Thermodynamics. According to the First Law of Thermodynamics, it is not possible to create or destroy energy, and so it is impossible to realize a *perpetuum motion machine of the first kind* able to continuously create its own energy. On the other hand, as stated by the Second Law of Thermodynamics, there is a restriction regarding the employment of energy that can be utilized only in a particular way. Therefore, it is impossible to realize a *perpetuum motion machine of the second kind* able to use the internal energy of only one heat reservoir. Regarding the latter, it would be ideally possible to realize this condition only if the external reservoir at a lower temperature is at zero Kelvin.

The exchange between heat and work in thermodynamic engines can be regarded as a conversion between a form of *degraded energy* at fixed *T*, the exchanged heat, and the work done. The adjective “degraded”, when associated with heat, underlines the impossibility of practically employing, in a complete way, this form of energy as, instead, occurs for potential or kinetic energy in mechanical systems. This is a significant distinction placing thermodynamics and mechanics on different footings.

On the other hand, Clausius developed the mechanical theory of heat, showing a contradiction between Carnot’s theorem [7] and the conservation of energy [8], and proposed another formulation of the Second Law of Thermodynamics. The Clausius statement [9,10,11] reads “*It is impossible to construct a refrigerator that, operating in a cycle, will produce no effect other than the transfer of heat from a lower-temperature reservoir to a higher-temperature reservoir”*. In other words, the transfer of heat from a higher-temperature reservoir to a lower-temperature reservoir is spontaneous; therefore, it has no cost in terms of work. Instead, the transfer of heat from a lower-temperature reservoir to a higher-temperature reservoir is possible only if, at the same time, work is done and this is what occurs for a refrigerator operating in a cycle. Apparently, the Clausius statement seems different from the Kelvin–Planck one, but it can be shown that the two statements are equivalent: by proving the violation of the Kelvin–Planck statement, the violation of the Clausius statement can be proven and vice versa. At the level of a deeper analysis: are there novel proofs of the equivalence of the two statements based on their fulfilment rather than on their violation? The question is subtle and still open and might potentially lead to new trends regarding the Second Law of Thermodynamics and to further important physical consequences.

A deeper understanding of the Second Law of Thermodynamics is gained by referring to its second classical formulation that is based on the introduction by Clausius in 1865 of a function of state *S*, called entropy [11,12]. As for the other functions of state, *S* depends only on the initial and final thermodynamic equilibrium states (the initial and final equilibrium states are a conditio sine qua non). In this second formulation, the relationship between the thermodynamic entropy *S* and the heat *Q* exchanged at a temperature *T* is established and can be regarded as the generalized Clausius inequality for closed thermodynamic systems, each of them constituted by the system itself and its local surroundings. For reversible processes, in infinitesimal form, it is *dS* = *δQ/T*, with *dS* being the infinitesimal entropy change or variation and *δQ* being the infinitesimal heat exchange. Taking into account the Kelvin–Planck statement and the definition of entropy, it can be stated that *S* quantifies the degraded energy at a given *T*. Importantly, for irreversible processes, real processes occurring in nature, *dS* is larger than *δQ/T* at a given temperature *T* of the surroundings, implying the presence of other sources of entropy.

A few years after Clausius coined the term entropy and stated its formulation of the Second Law of Thermodynamics, Maxwell, in 1871, raised a criticism of this version of the Second Law of Thermodynamics [13,14] by means of a thought experiment based on the so-called *Maxwell’s demon*, a tiny being who operates on the molecules of a gas contained in a box subdivided in two parts of equal volumes and at the same temperature. The demon opens the separation barrier, favoring the passage from the left to the right part of the box (and vice versa) for some of the molecules with specific kinematic features. The demon is also referred to as the temperature demon. Maxwell thus concluded that it is possible to transfer heat from a colder to a hotter part of a box without doing work, thus contradicting the Second Law of Thermodynamics. Later, several physical reasons about the impossibility of realizing this device were put forward, thus saving the Second Law of Thermodynamics, but it is still a not completely solved paradox and further arguments are expected.

Owing to the definition of entropy, the Second Law of Thermodynamics has been reformulated in rigorous mathematical and geometric terms by means of Carathéodory’s principle [5,15], stating “*In the neighborhood of any arbitrary initial state i of a physical system there exist neighboring states that are not accessible from i along quasi-static adiabatic paths*”. According to this principle, which has been rigorously proven, entropy results from a purely mathematical argument without requiring the use of engines and refrigerators. Initially, this principle appeared totally independent of the Kelvin–Planck and Clausius statements but it has been proved that it can be a direct consequence of these statements [16,17,18,19,20], and therefore, one can give up to this principle as independent of the other classical statements. However, a debate on this interesting topic might still be active. In this respect, the open question is as follows: is Caratheodory’s principle really redundant or does it add new insights to the comprehension and interpretation of the Second Law of Thermodynamics?

The strong advantage resulting from the introduction of the concept of entropy is the possibility to distinguish between *reversibility* and *irreversibility*. In this respect, ideal processes are reversible processes, while natural processes are irreversible processes. Reversible processes fulfil time reversal symmetry and irreversible processes break time reversal symmetry. However, the concepts of thermodynamic reversibility and irreversibility have a wider meaning with respect to the corresponding mechanical reversibility and irreversibility, which simply imply tracing back or not a particular phenomenon occurring in a mechanical system (physical, chemical, biological, etc.) by reversing time. Indeed, in thermodynamics, not only the thermodynamic system itself but also its local surroundings should be considered to define the “local” thermodynamic universe and the irreversibility. Other nonlocal portions of the surroundings make up part of the rest of the thermodynamic universe. Reversibility occurs only in ideal processes for which both the system and the local surroundings in the final state can be brought back to the initial state without producing any change in the rest of the thermodynamic universe. Irreversible processes are real processes resulting also in changes in the rest of the thermodynamic universe. In this language, changes are regarded as entropy variations. To quantify the distinction between reversible and irreversible processes, it is necessary to recall Clausius inequality for an internally cycle in a heat engine expressed as the closed integral of the infinitesimal heat *δQ* absorbed by a system from the reservoir at the temperature *T* [5]:

(1) $$\oint \frac{\delta Q}{T}\leq 0$$

with the sign = (<) referring to reversible (irreversible) processes.

The generalized Second Law of Thermodynamics for closed thermodynamic systems exchanging heat with the surroundings at temperature *T* can be written in integral form, starting from the Clausius inequality, as follows [5]:

(2) $$\Delta S\geq \int \frac{\delta Q}{T}$$

where Δ*S* = *S*_f_ − *S*_i_ > 0 is the change in entropy or entropy variation, with *S*_f_ (*S*_i_) being the entropy of the final (initial) state. Here, the equality holds for reversible processes, the inequality applies to both irreversible processes and quasi-static irreversible processes, namely slow processes having internal physical thermodynamic equilibrium characterized only by heat flow. The strict inequality can be converted into an equality, written as follows:

(3) $$\Delta S=\int \frac{\delta Q}{T}+\Delta S_{irr}$$

where Δ*S*_irr_ = *S*_irr f_ − *S*_irr i_ > 0 is the additional change in entropy or entropy variation related to irreversible processes from external and internal mechanical, thermal, and chemical sources, etc., with *S*_irr f_ (*S*_irr i_) being the related entropy of the final (initial) state. The determination of Δ*S*_irr_ is a formidable task that, if solved, would allow for quantifying the total entropy variation for irreversible processes in an exact way.

The formulation based on the definition of entropy paves the way to the finite form of the Second Law of Thermodynamics for isolated systems (*δQ* = 0), according to which Δ*S* ≥ 0 [5]. The entropy of an isolated thermodynamic system either remains constant for reversible processes or increases for irreversible processes, and the system evolves towards a global thermodynamic equilibrium. In practice, this corresponds to the principle of an increase in entropy, viz. the entropy of the thermodynamic “universe” (system + local surroundings) increases. By introducing the concept of entropy as a measure of disorder, it can be stated that the *“universe” passes from states of relative order to states of relative disorder*, marking the unidirectional nature of time and this statement can be extended to the real universe being regarded as an isolated thermodynamic system. The principle of an increase in entropy can be also applied to first-order phase transitions (e.g., solid–vapor), which are irreversible processes. It is true that the reverse transition can also occur (e.g., vapor–solid) but, even though the change in entropy at the transition temperature is the same with opposite sign, there are modifications in the composition passing from solid to vapor and vice versa, implying different changes in entropy both in the direct and reversed processes ascribed, in thermodynamic language, to the local surroundings. A similar argument can be applied to other first-order phase transitions and chemical reactions, implying an increase in entropy passing from reagents to products, an expression of chemical irreversibility, and, for example, to irreversible processes occurring in mechanical or thermal machines in the engineering field.

The introduction of the concept of *negative entropy* by Schrödinger in living systems [21] and of *negentropy* by Brillouin in information theory [22] as the tendency towards order and complexity has further deepened and widened the significance of the classical formulation of the Second Law of Thermodynamics. In this respect, the debate between syntropic phenomena (negative entropy) occurring ideally from the future to the past and implying a reversal of the time arrow in living systems, and entropic phenomena (positive entropy) [23] occurring in inanimate systems from the past to the future is still current, and novel trends and formulations are expected. The duality between syntropic and entropic phenomena would allow for regarding classical thermodynamics, when including both inanimate and living systems, also as a time-reversal branch of physics like classical mechanics. Owing to this duality, the analogy between thermodynamics and mechanics would be strengthened, opening new directions in classical physics.

An important and debated topic is also the classical relativistic form of the Second Law of Thermodynamics. There exists both a special relativity and a general relativity form. In special relativity thermodynamics, entropy is invariant with respect to the two inertial reference frames O and O’, the former at rest and the latter moving with uniform rectilinear motion at velocity v, viz. *S*_O_ = *S*_O’_. The Second Law of Thermodynamics in special relativity is the same as in the classical form (Equation (2)) in terms of its proper coordinates, while it depends on the Lorentz factor via the temperature passing to the general coordinates. If recurring to the proper coordinates and to a local observer, the Second Law of Thermodynamics can be written as a four-dimensional expression in terms of entropy density, not only in special relativity but also in general relativity with, in the latter case, the additional replacement of the ordinary divergence operator with the contracted covariant derivative [24]. However, even just focusing on the special relativity form of the Second Law of Thermodynamics, a question is still open because entropy invariance is true only assuming reversible and adiabatic heat exchanges without altering the internal state of the system integral with the reference frame O’ moving with velocity v. What would happen if this assumption was not anymore valid? It should be expected that *S*_O_ ≠ *S*_O’_, with the need to rewrite the classical relativistic form of the Second Law of Thermodynamics. And, what would happen when dealing with non-inertial systems of general relativity? The debate is still open.

The classical Second Law of Thermodynamics can be written in local form via the definition of a rate of entropy production *r*(*t*) = *dS*/*dt* or a rate of entropy density production *r*(*t*) = *ds*/*dt* with the entropy density *s* = *S*/*V* and with *V* being the volume [25]. *r*(*t*) expresses the entropy or entropy density time variation in isolated, closed, and open thermodynamic systems (generalized local form) including heat, matter, and chemical reactions contributions to the rate of entropy production (or rate of entropy density production) for the states of local thermodynamic equilibrium tending towards a global thermodynamic equilibrium.

Up until now, inspired by the formulation of a local form of the classical Second Law of Thermodynamics, quantum dynamic versions of the Second Law of Thermodynamics have been developed [13,26,27,28,29,30,31,32,33,34,35,36] using the Von Neumann quantum entropy *S*_VN_ applied to quantum open thermodynamic systems. However, novel quantum formulations of the Second Law of Thermodynamics still represent a challenging subject for basic and applied physics. Furthermore, a novel formulation could be derived by overriding the concept of entropy and could be based on the quantization of the exchanged heat or by introducing a quantum mechanics definition of work. Other formulations could take into account the role of quantum correlations and quantum entanglement in thermodynamics and their only apparent violation of the Second Law of Thermodynamics [37].

A combination of the quantum and relativistic formulations could also lead to a novel quantum relativistic form of the Second Law of Thermodynamics that would represent one of the new challenges of modern physics. One of the main question of this Special Issue is as follows: how would the entropy invariance employed in classical relativistic thermodynamics be described in a quantum relativistic formulation of the Second Law of Thermodynamics?

The aim of this Special Issue is to collect articles on new trends, ideas, and perspectives about unsolved and recent debated aspects of the Second Law of Thermodynamics in its classical, local, quantum, relativistic, and quantum relativistic formulations and about its related subjects. This allows for gaining a deeper understanding and advancement of the fundamental bases of this physical law, arousing curiosity about this in the scientific community, and stimulating the debate on modern thermodynamics. Applications are not restricted to the physical processes but also include chemical–physical, chemical, engineering, natural and life sciences processes. Philosophical implications of the Second Law of Thermodynamics are also accepted.

Original research manuscripts, review papers, and perspective/commentary articles on these important subjects are welcomed.
